# Developing a Framework Strategy for Supporting Radiographers in the Clinical Supervision of Radiography Students in Zambia: A Mixed Methods Study

**DOI:** 10.4314/ejhs.v30i6.15

**Published:** 2020-11

**Authors:** Osward Bwanga

**Affiliations:** 1 Midland Regional Hospital at Tullamore, Radiology Department, Co. Offaly, Ireland

**Keywords:** Clinical supervisor, clinical supervision, radiographer, radiography student, Zambia

## Abstract

**Background:**

Clinical supervisors of radiography students play a key role in the facilitation of practice-based learning. However, there is a scarcity of evidence-based strategies to support clinical supervisors. This study aimed at exploring the level of support required by radiographers in order to develop a framework strategy for supporting clinical supervisors of radiography students in Zambia.

**Methods:**

This study used an exploratory sequential mixedmethods approach. The qualitative phase was conducted first, and the findings were used to develop the questionnaire for the quantitative phase. The study population was radiographers working in the Lusaka and Copperbelt provinces of Zambia. For the first phase, data were collected from a purposive sample of 10 clinical supervisors of radiography students. For the survey, data were collected from 120 radiographers using a questionnaire. In the third phase, a group of experts validated the proposed framework using an online questionnaire. Qualitative data were analysed thematically and quantitative data using statistics.

**Results:**

Four support areas were identified: training and education in clinical supervision, clinical training resources, human resources and relationships, and quality assurance programmes related to clinical supervision. These findings informed the development of a support framework strategy for clinical supervisors.

**Conclusion:**

This study has revealed that clinical supervision of radiography students requires coordinated support from stakeholders: schools of radiography, professional body, and radiology and hospital management. It is anticipated that the developed framework, when implemented, will enhance the experiences of clinical supervisors and improve the quality of clinical education.

## Introduction

Teaching in the clinical environment is an integral part of radiography education which allows students to develop their competence (1). Radiographers who supervise students are called clinical supervisors and constitute a vital resource in this development (2). To perform this role efficiently and effectively, clinical supervisors should receive adequate support from stakeholders (1–3). The school of radiography has a responsibility to collaborate with clinical departments (2,3). Professional bodies have a duty of providing continuing professional development (CPD) (4–6). The radiology and hospital managements have a responsibility to provide training resources (1,3).

Most of the strategies for supporting clinical supervisors documented in the literature are from the nursing profession: protected time, support groups, and training in supervision (7–10). However, a review by the Royal College of Nursing (11) found that the issue of support for clinical supervisors in nursing education is poorly addressed. Nursing studies have also revealed inadequate support for clinical supervisors from stakeholders (9,12,13).

In Zambia, there is a lack of evidence-based strategies for supporting radiographers in the clinical supervision of radiography students. The researcher, being a clinical educator, had also heard radiographers expressing concern about and dissatisfaction with the support received from stakeholders in their roles as clinical supervisors. The aim of this study was to explore the level of support required by radiographers in order to develop a framework strategy for supporting clinical supervisors in Zambia.

## Methods

This study employed a mixed-methods research approach using an exploratory sequential design. Qualitative data were collected and analysed in the first phase and the findings were used towards the development of a questionnaire for the quantitative phase (14,15).

**Study population and sampling**: The study population comprises of radiographers working in the Lusaka and Copperbelt provinces of Zambia. The first phase included a purposive sample of ten clinical supervisors working at two teaching hospitals. The criteria for recruitment were radiographers with more than two years' experience in the supervision of students as per the eligibility criteria of a clinical supervisor (16,17). The sample included chief radiographers (n=2), principal radiographers (n=2), senior radiographer (n=1) and radiographers (n=5). In the second phase, the entire accessible population (N=160) was included due to the small population.

**Data collection and analysis**

***Phase 1: Qualitative approach*** Data were collected in July 2018, using semi-structured interviews from 10 participants. Interviews were audio recorded. The average duration of each interview was 25 minutes.

The audio recordings were transcribed verbatim and data analysed thematically using the Braun and Clarke (18) five-phase thematic framework: familiarisation with data, generating initial codes, searching for themes, reviewing themes, and naming themes. Three themes and seven sub-themes emerged, and these informed the development of a questionnaire for the second phase (14,15,19).

***Phase 2: Quantitative approach***

Data were collected using a self-administered questionnaire between October and December 2018. The questionnaire consisted of five questions on respondents' gender, position, province, age, education and experience, and 8 statements on the supports required by clinical supervisors. Respondents were requested to indicate “Yes” if they agreed with the statement or “No” if they disagreed. An option of “other” was included to allow respondents to write suggestions in their own words.

Data entry and analysis took place using Microsoft Excel for Windows 2016 and SPSS software version 25. Quantitative data were analysed using descriptive statistical methods; namely, frequencies, percentages and tables.

**Ethical considerations**: This study was part of a larger research on strategies to support radiographers in the clinical supervision of radiography students in Zambia. The study commenced after obtaining ethical approval from the Tropical Diseases Research Centre (TDRC) Research Ethics Committee. Permission was also obtained from all the heads of the X-ray departments at the study sites. The details of the study were disclosed to each participant and confidentiality and anonymity were assured. In the first phase, informed consent was obtained from each participant, whereas in the second phase, the completion and returning of the questionnaire constituted consent (14).

## Results

**Phase 1: Qualitative approach**

Three themes and seven sub-themes emerged following data analysis (Table 1).

**Table 1:** Suggested support strategies to enhance radiographers in their role as clinical supervisors of radiography students

Theme	Sub-themes
**Theme1:** Education and training	Establishment of a clinical supervision training programme Provision of CPD learning activities related to clinical supervision
**Theme 2:** Equipment maintenance and training resources	Prompt repairs of X-ray equipment Publication of clinical supervision guidelines Provision of more consumables during students' clinical placement
**Theme 3:** Designated radiographer (link clinical tutor) and incentives	Appointment of a designated radiographer in each X-ray department Provision of incentive for clinical supervisors

**Theme 1: Education and training**

Two suggestions related to education and training were identified: the establishment of a clinical supervision training programme and the provision of CPD.

***Sub-theme 1: Establishment of a clinical supervision training programme***

Six participants suggested the establishment of a clinical supervision training programme:

“I would like the schools of radiography to provide me with training to enhance my supervision skills”. (Participant 6)

***Sub-theme 2: Provision of CPD***

Three participants suggested the provision of CPD related to clinical supervision:

“The Radiological Society of Zambia (RSZ) could offer CPD in clinical supervisi on”. (Participant 9)

**Theme 2: Equipment maintenance and training resources**

This theme yielded three sub-themes: prompt repair of X-ray equipment, the publication of guidelines and the provision of more consumables during students' placements.

***Sub-theme 1: Prompt repairs of X-ray equipment***

The prompt repairs of X-ray equipment was a support area suggested by three participants:

“Fix broken imaging equipment on time, so that I can continue with students' training”. (Participant 4)

Radiographers attributed the frequent breakdown of X-ray equipment due to a lack of maintenance.

***Sub-theme 2: Publication of clinical supervision guidelines***

Five participants suggested the publication of guidelines to guide radiographers in the facilitation of practice-based learning:

“The college should provide us with supervision guidelines”. (Participant 1)

***Sub-theme 3: Provision of adequate Consumables***

The provision of adequate resources during students' placement was another support area suggested by four participants:

“There is a need to improve the supply of consumables such as X-ray films and contrast media agents because there is a waste of resources during training”. (Participant 2)

**Theme 3: Designated radiographers and incentives for clinical supervisors**

This theme had two sub-themes: appointment of designated radiographers and the provision of incentives for clinical supervisors.

***Sub-theme 1: Appointment of a designated radiographer in each X-ray department***

Three participants suggested the appointment of a dedicated radiographer to act as a link between schools of radiography and placement site:

“There is a need to create a link between the schools of radiography and the radiographers involved in supervision by appointing link clinical tutors”. (Participant 10)

***Sub-theme 2: Provision of incentives for clinical supervisors***

Seven participants suggested some form of material reward for the extra duties' radiographers undertake in clinical supervision:

“There is also a need for radiographers who teach students to receive teaching allowances”. (Participant 3)

**Phase 2: Quantitative approach**

Out of 160 administered questionnaires, 120 were completed and returned, giving a response rate of 75%. Table 2 shows the demographic data of respondents.

**Table 2:** Demographic characteristics of respondents (N=120)

Characteristic	Category	Proportion	Percentage (%)
**Gender**	Female	53	44.2
	Male	67	55.8
**Province**	Lusaka	75	62.5
	Copperbelt	45	37.5
**Highest level of** **education**	Diploma	81	67.5
	Bachelors	33	27.5
	Masters	6	5.0
**Work experience**	1–5 years	41	34.17
	6–14 years	47	39.17
	15 years and above	32	26.67

**Education and training**: The provision of education and training had two suggestions. Table 3 shows that the majority, N= 119 (99.2%) and N=117 (97.5%), of the respondents, indicated the establishment of a clinical supervision training programme and provision of CPD respectively, as one of the support strategies required by clinical supervisors.

**Table 3:** Scores on the suggested support strategies that could enhance radiographers in the clinical supervision of radiography students (N=120)

Suggestion	Yes	No
Establishment of a clinical supervision training programme	119(99.2%)	1(0.8%)
Continuing professional development (CPD) on clinical supervision	117(97.5%)	3(2.5%)
Prompt repairs of X-ray equipment	113(94.2%)	7(5.8%)
Publication of clinical supervision guidelines	114(95.0%	6(5.0%)
Provision of more consumables during students' clinical placement	115(95.8%)	5(4.2%)
Appointment of a designated radiographer	103(85.8%)	17(14.2%)
Provision of incentives to clinical supervisors	111(92.5%)	9(7.5%)
Establishment of educational audit programme for placement sites	92(76.7%)	28(23.3%)

**Equipment maintenance and training resources**: Table 3 shows that most, N=113 (92.8%), of the respondents indicated that the prompt repair of X-ray equipment would be beneficial support. A majority, N=114 (95.0%) and =115 (95.8%), of the respondents, also indicated the publication of guidelines and provision of more consumables during students' placement respectively, would be some of the support strategies for clinical supervisors.

**Designated radiographers and incentives**: Table 3 shows that more than three-quarters, N=103 (85.8%), of the respondents concorded the suggestion of the appointment of a designated radiographer in each X-ray department to link the clinical site with the schools of radiography. The suggestion of incentives was also proposed by most N=111 (92.5%), of the respondents who indicated the need for rewarding radiographers involved in clinical supervision.

**Educational audit programme**: Table 4 shows that most, 92(76.7%), of the respondents suggested the establishment of an educational audit programme at the clinical training sites.

**Table 4:** Validators' demographic information (N=6)

Occupation	Highest qualification	Professional experience
Radiography lecturer	MSc Radiography	21 years
Radiography lecturer	MSc Radiography	19 years
Chief radiographer	MSc Radiography	20 years
Chief radiographer	MSc Radiography	17 years
Clinical tutor	MSc Radiography	16 years
Radiological Society of Zambia official	BSc Radiography	16 years

## Discussion

This study revealed a lack of appropriate knowledge and skills for effective clinical supervision amongst radiographers due to the non-availability of a clinical supervision training programme. Similar findings were identified in studies conducted by Sutton (20) and Lee (21) which reported a lack of knowledge and skills about educational principles amongst radiographers who supervise students. Poor practices in clinical supervision may result from these findings (7,22). The strategy of establishing a clinical supervision training programme has been researched in nursing education. For example, Neshuku and Justus (8) developed a course for clinical supervisors in Namibia. The findings of this nursing study determined that clinical supervisors improved their knowledge and skills of facilitation of practice-based learning. The present study identified schools of radiography as responsible for developing and providing clinical supervision training to clinical supervisors.

A lack of CPD related to clinical supervision came forward from this study. This is an interesting finding because, in a study conducted in South Africa by Mung'omba and Botha (23), radiographers failed to identify clinical supervision as an additional core competency required by radiographers. This strategy would ensure that clinical supervisors are kept up-to-date with developments in the clinical supervision of students. In this study, the RSZ was identified as being responsible for providing formal CPD activities.

A lack of communication and coordination between the schools of radiography and clinical training sites came through as a challenge in clinical supervision. For this reason, radiographers suggested the appointment of a designated radiographer to be responsible for clinical supervision. This finding is in line with Walsh (7) who advised the need for a link clinical tutor responsible for supporting supervisors and students, managing teaching aids, communication, and putting in place a programme on quality assurance. In other words, a designated radiographer will act as a link and would help to improve the communication between the schools of radiography and clinical sites.

This study found a lack of incentives as an inhibiting factor to clinical supervision. Radiographers suggested the need for incentives for clinical supervisors in the form of a teaching allowance and education sponsorship opportunities. This finding is in line with a study carried out by Eta et al. (24) in Cameroon, where clinical educators found it challenging to supervise nursing students effectively without incentives.

This study found a lack of clinical supervision guidelines from the schools of radiography and RSZ. This finding is in contrast with the Society of Radiographers of the UK, which has issued several guidelines to assist radiographers in the facilitation of radiography students' learning (1,3,25–27). In light of this, there is a necessity to publish guidelines to guide radiographers in the facilitation of learning.

This study revealed poor maintenance and servicing of imaging equipment. This finding agrees with the Ministry of Health Strategic Plan of 2017 to 2021 (28), which attributed the frequent faulty and breakdown of imaging equipment due to a lack of preventive maintenance programmes in Zambia. Preventive maintenance and servicing minimises the financial costs of a total breakdown of imaging equipment and disruption of services (29). Thus, a need for a national maintenance and servicing programme in Zambia (28). This support would prevent disruption to radiology services and training of radiography students.

Radiographers expressed concerns about a lack of consumables such as X-ray films and contrast media agents. This finding agrees with a study conducted in Rwanda by Ondari et al. (30) which found an inadequate supply of consumables during radiography students' placements. The resource-constrained learning environment is also a challenge in the nursing profession in Africa (8,24,31,32). There is a need to provide more consumables by hospital management during students' placements.

This study revealed a lack of educational audits of clinical training sites. Walsh (7) states that audits are beneficial in identifying areas in which clinical supervisors need help and support to maintain, improve and develop a quality learning environment. There is a necessity to develop and conduct periodic audits of clinical training sites. The schools of radiography and RSZ were identified as being responsible for the establishment of educational audits.

**Figure 1:**
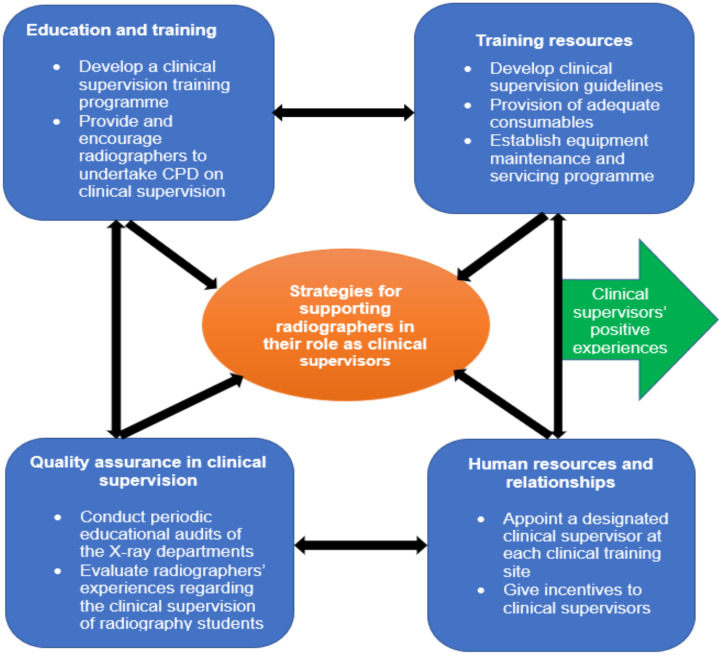
Proposed framework strategy for supporting clinical supervisors

**Proposed strategy framework for supporting clinical supervisors**: The overarching aim of this study is to develop a framework strategy for supporting clinical supervisors in Zambia. In line with the aim and study findings, the following framework was drafted and proposed (Figure 1).

**Validation of the proposed strategy framework**: A group of experts (N=6) was requested to validate the proposed framework strategy. Table 4 (see above) outlines the description of demographic information of validators.

Data were collected using an online questionnaire in February 2019. The questionnaire was develop based on the seven criteria identified from the literature: clarity, credibility, relevance, efficiency, sustainability, implementability and impact (23,33). The questionnaire was created and administered via www.surveymonkey.com. Each validator was requested to rate the framework on a five-point Likert scale from “strongly agree” to “strongly disagree” if it met the adopted criteria, and to comment on each criterion where necessary. The scores are presented in Table 5.

**Table 5:** Scores of validators of the proposed strategy framework (N=6).

Criteria	Strongly agree	Agree	Undecided	Disagree	Strongly disagree	Total
**Clarity**- The strategy framework is simple and clear to understand	3	3	0	0	0	6
**Credibility**- If implemented, the strategy framework can be trusted to enhance the experiences of clinical supervisors	2	4	0	0	0	6
**Relevance**- The strategy framework is appropriate for clinical supervisors	2	3	0	1	0	6
**Efficiency**- The strategy framework is economically sound in the environments where it will be implemented	0	6	0	0	0	6
**Implementable**- The strategy framework can be implemented in the X-ray departments	3	2	1	0	0	6
**Impact**- The strategy framework can result in positive experiences for clinical supervisors	4	2	0	0	0	6
**Sustainability**- The strategy framework can have long-term benefits in supporting clinical supervisors	3	3	0	0	0	6

The first statement was on the clarity of the proposed strategy framework. The result from Table 5 shows that the validators who “strongly agreed” and “agreed” to the statement were equal. One group member commented that:

“*The proposed framework is easy to understand”. (RSZ official)*

The results portray that all validators believed that the proposed framework is clear and simple to understand.

The second statement asked expert group members to indicate whether the proposed strategy framework was creditable. Table 5 reveals that two validators “strongly agreed” and four “agreed” to the statement. The results mean that all validators believed that the proposed framework is trustworthy, and when implemented, could enhance the experiences of clinical supervisors.

The third statement focused on the relevance of the proposed strategy framework. Data presented in Table 5 reveals that two validators “strongly agreed” and three “agreed” to the statement. However, one validator disagreed:

“The framework may be developed, but clinical supervisors also need training”. (RSZ official)

This concern was taken into consideration when developing the framework and included under education and training. The results show that most of the validators were satisfied that the framework was relevant for supporting clinical supervisors.

The fourth statement concentrated on the efficiency of the proposed strategy framework. Table 5 indicates that all validators agreed with the statement that the proposed framework is economically sound in the learning environments where implementation will happen.

In a fifth statement, expert group members indicated their opinion regarding the implementability of the strategy framework. Table 5 shows that the validators who “strongly agreed” and “agreed” to the statement were equal. The overall results indicate that all validators believed that the proposed framework is implementable in the clinical sites.

The sixth statement endeavoured to elicit validators' opinions on the impact of the proposed strategy framework. The results show that four validators “strongly agreed” and two “agreed” to the statement. This finding suggests that all validators were satisfied that the framework could result in positive experiences for clinical supervisors.

The final statement dealt with the sustainability of the proposed strategy framework. Table 5 shows that the validators who “strongly agreed” and “agreed” to the statement were equal. Although all validators believed that the proposed strategy framework could have long-term benefits in supporting clinical supervisors, one had a concern:

“There is a need to look at the role of the training institution concerning clinical supervision, e.g. are the training institutions overenrolling and what is the impact of such to clinical supervisors”. (Clinical tutor)

This concern was considered during the development of the framework and covered under quality assurance. Evaluation of radiographers in clinical supervision will reveal the impact of over enrolment of students.

The comments from validators were not significant enough to make any amendment to the framework. Therefore, the developed framework still maintains the four support areas: training and education, training resources, human resources and relationships, and quality assurance programmes (Figure 1, see above).

In conclusion, the developed strategy framework, if implemented, would enhance the experiences of clinical supervisors of radiography students in Zambia. At the time of this study, the researcher was not aware of any framework that guided stakeholders in supporting clinical supervisors of radiography students and this study has accomplished the task of bridging the gap which existed in the literature. Whilst addressing most of the strategies suggested in the framework at the local level, there are others which require intervention at a higher policy level, the Ministry of Health of Zambia. A further study, measuring the impact of the strategies on the experiences of clinical supervisors, following framework implementation, may be undertaken.
